# HIV testing and ART initiation among partners, family members, and high-risk associates of index clients participating in the CommLink linkage case management program, Eswatini, 2016–2018

**DOI:** 10.1371/journal.pone.0261605

**Published:** 2021-12-20

**Authors:** Daniel Williams, Duncan MacKellar, Makhosazana Dlamini, Johnita Byrd, Lenhle Dube, Phumzile Mndzebele, Sikhathele Mazibuko, Trong Ao, Ishani Pathmanathan, Alysha Beyer, Caroline Ryan

**Affiliations:** 1 Division of Global HIV and TB, Center for Global Health, U.S. Centers for Disease Control and Prevention, Atlanta, Georgia, United States of America; 2 Eswatini Country Program, Population Services International, Mbabane, Eswatini; 3 ICF International, Atlanta, Georgia, United States of America; 4 National AIDS Programme, Eswatini Ministry of Health, Mbabane, Eswatini; 5 Eswatini Country Office, U.S. Centers for Disease Control and Prevention, Mbabane, Eswatini; 6 Abt Associates Inc, Rockville, Maryland, United States of America; University of Washington, UNITED STATES

## Abstract

To help diagnose and initiate antiretroviral therapy (ART) for ≥95% of all persons living with HIV (PLHIV), the World Health Organization (WHO) recommends offering HIV testing to biological children, and sexual and needle-sharing partners of all PLHIV (index-client testing, ICT). Many index clients, however, do not identify or have contactable partners, and often substantially fewer than 95% of HIV-positive partners initiate ART soon after index testing. To help improve early HIV diagnosis and ART initiation in Eswatini (formerly Swaziland), we implemented a community-based HIV testing and peer-delivered, linkage case management program (CommLink) that provided ICT as part of a comprehensive package of WHO recommended linkage services. CommLink was implemented June 2015 –March 2017 (Phase I), and April 2017 –September 2018 (Phase II). In addition to biological children and partners, HIV testing was offered to adult family members (Phases I and II) and high-risk associates including friends and acquaintances (Phase II) of CommLink index clients. Compared with Phase I, in Phase II proportionally more CommLink clients disclosed their HIV-infection status to a partner or family member [94% (562/598) vs. 75% (486/652)], and had ≥1 partners, family members, or high-risk associates (contacts) tested through CommLink [41% (245/598) vs. 18% (117/652)]. Of 537 contacts tested, 253 (47%) were HIV-positive and not currently in HIV care, including 17% (17/100) of family members aged <15 years, 42% (78/187) of non-partner family members aged ≥15 years, 60% (73/121) of sexual partners, and 66% (85/129) of high-risk associates. Among 210 HIV-positive contacts aged ≥15 years who participated in CommLink, nearly all received recommended linkage services including treatment navigation (95%), weekly telephone follow-up (93%), and ≥3 counseling sessions (94%); peer counselors resolved 76% (306/404) of identified barriers to care (e.g., perceived wellness); and 200 (95%) initiated ART at a healthcare facility, of whom 196 (98%) received at least one antiretroviral refill before case-management services ended. To help countries achieve ≥90% ART coverage among all PLHIV, expanding ICT for adult family members and high-risk associates of index clients, and providing peer-delivered linkage case management for all identified PLHIV, should be considered.

## Introduction

As part of the 95-95-95 initiative to control the HIV epidemic, countries in sub-Saharan Africa and elsewhere aim to initiate and retain ≥90% of all persons living with HIV (PLHIV) on antiretroviral therapy (ART) [1]. To help meet this 90% target, the World Health Organization (WHO) recommends offering HIV testing services (HTS) to biological children, and sexual and needle-sharing partners of all PLHIV (index-client testing, ICT) [2]. Accordingly, all HIV testing programs supported by the United States President’s Emergency Plan for AIDS Relief (PEPFAR) are expected to offer ICT for biological children and partners of all newly diagnosed PLHIV and link at least 95% of those who test HIV-positive to immediate ART [3].

Many index clients, however, do not identify or have partners that can be contacted, and often substantially fewer than 95% of HIV-positive partners initiate ART soon after index testing [4–16]. As suggested by studies of PLHIV diagnosed in community settings, HIV-positive partners who are not seeking healthcare services when testing may be particularly vulnerable to one or more barriers to care (e.g., perceived wellness), and many may need linkage services to initiate ART soon after diagnosis [2, 3, 17–20]. In addition to biological children and partners, index clients may also know others who might benefit from testing or receiving ART-linkage services, including adult, non-partner family members, and friends or acquaintances at high HIV risk or who are known to have defaulted from HIV care (high-risk associates).

Information on ICT outcomes for adult, non-partner family members and high-risk associates, and ART initiation among PLHIV who receive recommended linkage services after index testing, might help improve ICT case-finding and linkages to ART. This information may be particularly important for countries approaching 95-95-95 that need efficient strategies to identify and initiate ART on the remaining, potentially most difficult to reach PLHIV such as young adults aged 15–24 years and men [1–3].

To help improve early HIV diagnosis and ART initiation in Eswatini, we implemented a community-based HTS and peer-delivered, linkage case management program (CommLink) that provided ICT as part of a comprehensive package of U.S. Centers for Disease Control and Prevention (CDC) and WHO recommended HTS and linkage services [2, 3, 21, 22]. Eswatini has an estimated HIV prevalence of 27% among residents aged 15–49 years and was the first sub-Saharan Africa country to achieve 95-95-95 in 2020 [23]. Because of the diminishing yield of community-outreach HTS to identify undiagnosed PLHIV during the first phase of CommLink, ICT services were modified to include high-risk associates during the second phase of program expansion.

This paper reports on the uptake of CommLink ICT services and test outcomes of partners, family members, and high-risk associates (contacts) of CommLink clients. For those contacts and community-outreach clients who tested HIV-positive, we also summarize receipt of recommended linkage services, ART-initiation at healthcare facilities, and the types of barriers to care that peer counselors identified and helped resolve by the end of case management.

## Methods

CommLink methods have been described previously [24, 25]. Briefly, CommLink was implemented by Population Services International (PSI) in Hhohho, Manzini, and Lubombo regions of Eswatini in two phases: Phase I, June 2015 –March 2017, and Phase II, April 2017 –March 2018. Case management was completed for the last Phase II client on 14 September 2018. In Phase I, CommLink was implemented by two outreach HTS teams, each operating with a modified van with two rooms (mobile unit) in rural and urban Tinkhundla (similar to districts) in Hhohho and Manzini regions [24]. In Phase II, CommLink expanded to four mobile-unit teams and provided HTS and linkage services in three of the four regions of Eswatini [25]. In both phases, each team included two or three HTS counselors, three or four HIV-positive expert-client (peer) counselors, and a nurse. Regions and Tinkhundla where mobile-unit teams provided services were selected based on country operational plans, available resources, and testing needs determined in collaboration with regional health management teams of the Eswatini Ministry of Health and CDC.

### Target population and outreach testing

In both phases, the CommLink target population was undiagnosed PLHIV residents, particularly those aged 15–29 years and men, two groups estimated to have the lowest diagnostic and ART coverage in Eswatini [23]. To reach the target population, outreach HTS was conducted at work sites (e.g., mines, industrial areas, cattle dip tanks), social venues (e.g., bars and clubs), high-volume locations (e.g., near grocery stores and bus ranks), and community events (e.g., sport or holiday events). Tents and mobile units were used to provide private settings for testing, and pre- and post-test counseling. For some events, community mobilization was also conducted using disc jockeys, music and public announcements, and outreach staff to increase demand for testing. HTS was conducted in accordance with national rapid HIV testing guidelines: the screening test was Determine, and the second test, Uni-Gold, was conducted if Determine was reactive [26]. Clients with reactive results for both tests were considered HIV positive, provided confidential post-test counseling, and introduced to peer counselors who explained and assessed eligibility for linkage case management.

### Linkage case management

In both phases, consenting PLHIV who were newly or previously HIV diagnosed, had not received HIV care in the prior 90 days, and were referred to healthcare facilities in the region of HIV diagnosis or in regional boarder zones were eligible for CommLink linkage case management. Clients who were not eligible to receive case management were provided standard referrals in accordance with national guidelines [26]. Linkage case management during both phases included peer-delivered counseling and psychosocial support, escort and treatment navigation at healthcare facilities, weekly telephone calls, and at least two follow-up face-to-face counseling sessions focused on disclosure and ICT, and identifying and resolving barriers to HIV care [24, 25]. CommLink peer counselors were HIV-positive, ART-adherence counselors trained and certified by the Eswatini Ministry of Health. Peer counselors strived to appoint and meet their clients at healthcare facilities within 7 days of diagnosis. As part of treatment navigation, they met and stayed with their client for the duration of at least their first facility visit, provided psychosocial and informational support, and ensured that they understood the stations and sequence of HIV care [24, 25]. Participating clients were provided case management through enrollment in HIV care and at least the first antiretroviral refill if initiated on ART for up to 90 days [24, 25].

### Index client testing

During follow-up calls and face-to-face counseling sessions, CommLink peer counselors encouraged disclosure to and HIV testing of partners and family members when safe and appropriate. In both phases, ICT was offered to main sexual partners and all available family members including biological children and non-partner adults. In Phase II, peer counselors were re-trained on the importance of helping clients disclose their status to partners and family members when safe and appropriate, and ICT contacts were expanded to include high-risk associates and non-main partners with whom clients had sex in the past six months. High-risk associates were defined as persons other than sexual partners and family members (e.g., friends) known by clients to have defaulted from HIV care or who might benefit from HTS. For consenting clients who wished to use ICT services, peer counselors elicited the names, location, and phone numbers (if available) of contacts, and decided with index clients whether to offer HTS to contacts with or without the index client’s involvement. Unless specifically permitted by their clients, HTS and peer counselors never informed contacts of the identity of index clients.

### Data collection, management, and analyses

In both phases, peer counselors recorded on standardized forms uptake of specific linkage services; up to 13 different barriers to HIV care (e.g., perceived wellness, fear of stigmatization, high costs) that were identified and resolved during client-centered counseling; client disclosure of HIV status, acceptance of ICT, and test outcomes of contacts who were reached and consented to HTS; and enrollment-in-care, ART-initiation, and antiretroviral-refill outcomes among all clients who received linkage case management, including those identified through ICT. Identified barriers were recorded as “resolved” when peer counselors believed that they no longer interfered with or prevented early enrollment or retention in HIV care. Enrollment in care, defined as having received HIV clinical services at least once at a healthcare facility, and ART initiation and receipt of ≥1 antiretroviral refills, were recorded from patient medical records. All data were double-entered into CSPro v5.0, routinely evaluated for completeness and logic consistency, and when needed, cleaned using source records. Outcomes are reported as percentages for nominal and ordinal variables, and as medians and interquartile (IQR) ranges for ratio-scaled variables. Prevalence estimates with 95% confidence intervals and statistical tests for group differences were not calculated because CommLink clients are the program population of interest. All data were analyzed using SAS 9.4 (SAS Institute Inc., Cary, NC, USA).

### Human subjects

CommLink was approved by the Eswatini Ministry of Health and anonymized program data were submitted to and analyzed at CDC under an approved non-research project determination. All adult clients and parents or legal guardians of children aged <15 years who participated in CommLink provided oral informed consent for HTS and to receive follow-up linkage services in accordance with Eswatini national guidelines [26].

## Results

### Participation

**Fig 1:**
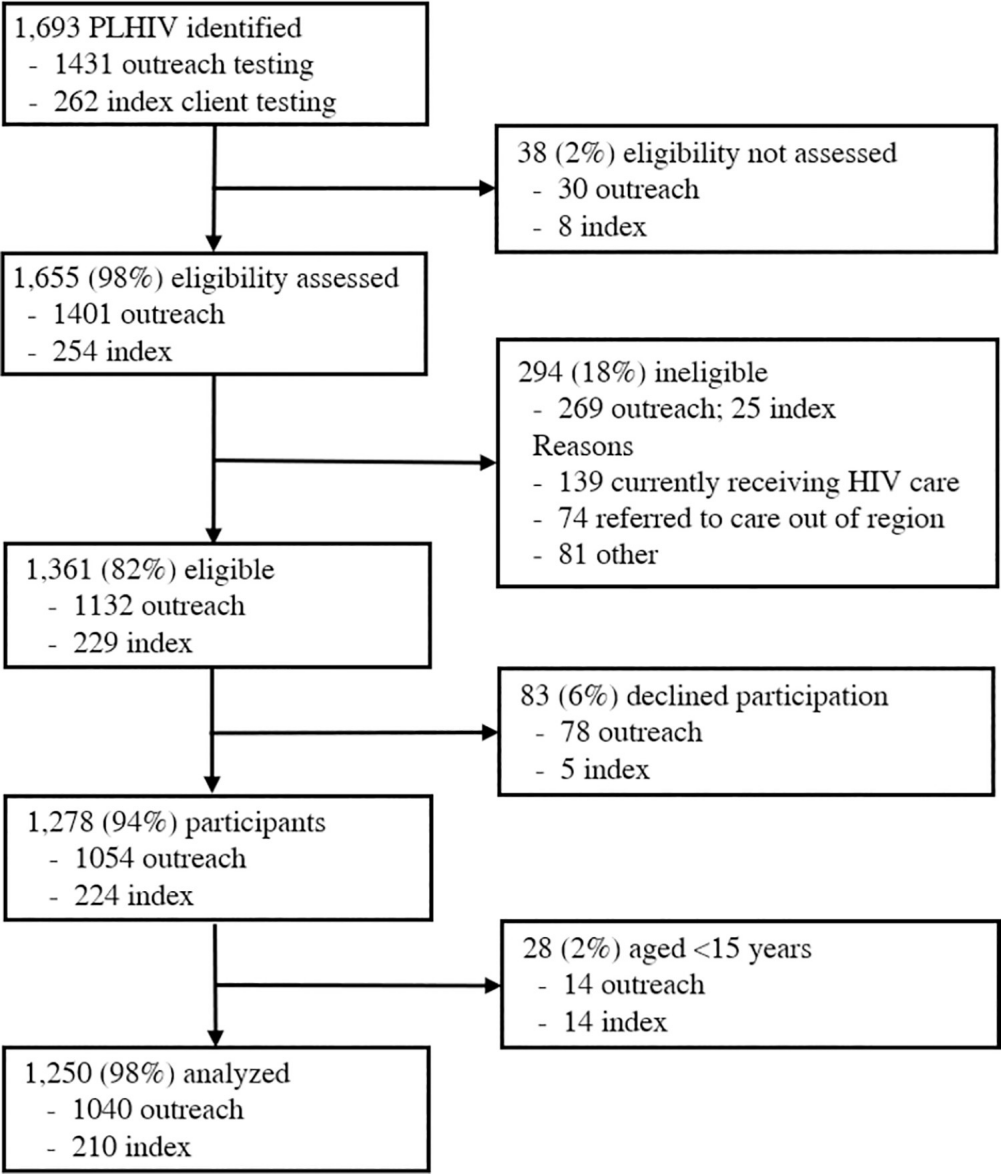
CommLink outreach and index client testing participation cascade.

From June 2015 through March 2018, 1,693 PLHIV were identified during CommLink outreach HTS (n = 1431) and ICT (n = 262) events. Of these, 1,655 (98%) were screened for eligibility and 294 were ineligible (Fig 1). Most ineligible PLHIV were currently in HIV care (47%), or were referred to a healthcare facility in another region of Eswatini (15%) or in another country (10%). Of 1,361 eligible clients, 1,278 (94%) consented to participate. Among consenting clients, analyses were restricted to 1250 (98%) adults aged ≥15 years, including 1040 and 210 identified through outreach HTS and ICT, respectively (Fig 1). The 1,250 PLHIV received linkage case management services over a median of 59 days (IQR: 43–79 days). Most clients were male, ≤34 years of age, newly HIV diagnosed, and single (Table 1).

**Table 1:** HIV testing of ≥ 1 sexual partners, family members, and high-risk associates (contacts) of CommLink clients, by program phase and client characteristics, Eswatini, June 2015 –September 2018.

	CommLink Clients							
Characteristic	All Clients^a^	≥1 Contacts	Partner(s) not	Partner	≥1 Family	Fam. Member	Phase II^e^	Associate
		Tested	in HIV Care	Tested	Members	Tested		Tested
	n (%)	n (%)^b^	n (%)^b^	n (%)^c^	n (%)^b^	n (%)^d^	n (%)	n (%)^f^
**Total**	1,250	362 (29)	668 (53)	119 (18)	1,158 (93)	187 (16)	598	111 (19)
**Phase**								
I (06/2015-03/2017)	652 (52)	117 (18)	294 (45)	54 (18)	561 (86)	77 (14)	--	--
II (04/2017-09/2018)	598 (48)	245 (41)	374 (63)	65 (17)	597 (100)	110 (18)	598 (100)	111 (19)
**Sex**								
Male	699 (56)	174 (25)	341 (49)	60 (18)	641 (92)	86 (13)	288 (48)	51 (18)
Female	551 (44)	188 (34)	327 (59)	59 (18)	517 (94)	101 (20)	310 (52)	60 (19)
**Age group (years)**								
15–24	207 (17)	64 (31)	134 (65)	17 (13)	199 (96)	36 (18)	115 (19)	24 (21)
25–34	541 (43)	144 (27)	311 (57)	55 (18)	496 (92)	71 (14)	248 (41)	39 (16)
>34	502 (40)	154 (31)	223 (44)	47 (21)	463 (92)	80 (17)	235 (39)	48 (20)
**HIV status**								
Newly diagnosed	727 (58)	184 (25)	422 (58)	76 (18)	655 (90)	91 (14)	283 (47)	42 (15)
Previously diagnosed	523 (42)	178 (34)	246 (47)	43 (17)	503 (96)	96 (19)	315 (53)	69 (22)
**Marital status** ^**g**^								
Cohabitating with 1 partner	409 (33)	125 (31)	216 (53)	57 (26)	372 (91)	62 (17)	174 (29)	30 (17)
Widowed/separated/divorced	43 (3)	14 (33)	12 (28)	0 (0)	41 (95)	10 (24)	25 (4)	5 (20)
Single	705 (56)	204 (29)	395 (56)	54 (14)	659 (93)	107 (16)	366 (61)	71 (19)

Contacts, sexual partners, non-partner family members, or high-risk associates of CommLink clients; high-risk associates, friends, coworkers, acquaintances etc. known by index clients to have defaulted from HIV care or who might benefit from HIV testing.

^a^Tested HIV-positive in community settings, reported not receiving HIV care in the prior 90 days, including ART, and consented to receive follow-up linkage case management services.

^b^% denominator = all clients.

^c^% denominator = clients with ≥1 partners not in HIV care.

^d^% denominator = clients with ≥1 family members.

^e^CommLink expanded ICT for high-risk associates of index clients, and increased from two to four mobile HTS outreach teams to provide services throughout three of the four regions of Eswatini.

^f^% denominator = Phase II clients.

^g^93 (7%) clients had missing marital status.

### Disclosure and uptake of index testing

Before case management services ended, 1,048 (84%) clients disclosed their HIV status to ≥1 partners or family members. Among 668 clients with at least one partner who was not in HIV care, 119 (18%) had ≥1 partners tested through CommLink; 187 (16%) of 1,158 clients with family members had ≥1 family members tested; and 111 (19%) of 598 Phase II clients had ≥1 associates tested. Overall, 362 (29%) clients had ≥1 contacts who tested for HIV through CommLink. Proportionally more clients who were older or were co-habitating with a partner had a partner who tested through CommLink, and proportionally more previously than newly diagnosed clients had an associate who tested (Table 1). Compared with Phase I, in Phase II proportionally more clients disclosed their HIV-infection status to a partner or family member [94% (562/598) vs. 75% (486/652)], identified ≥1 partners (63% vs. 45%) or family members (100% vs. 86%) who might be available for testing, and had ≥1 contacts tested through CommLink (41% vs. 18%).

### Index testing outcomes

Of 537 contacts tested, 262 were HIV-positive, of whom 253 (47% of 537) had not received HIV care in the prior 90 days (out-of-care), including 17% among children, 42% among family members aged ≥15 years, 60% among sexual partners, and 66% among high-risk associates (Table 2). Of these 253 HIV-positive contacts, 113 (45%) received an HIV diagnosis for the first time (newly HIV diagnosed). The percentage of tested contacts who were HIV-positive out-of-care increased from 27% (16% newly diagnosed) in Phase I to 58% (24% newly diagnosed) in Phase II (Table 2). Of HIV-positive out-of-care contacts, the percentage who had been previously diagnosed increased from 42% (22/52) in Phase I to 59% (118/201) in Phase II. In Phase II, of 201 HIV-positive out-of-care contacts, 147 (73%) were either non-partner family members aged ≥15 years (n = 62, 31%) or high-risk associates (n = 85, 42%).

**Table 2:** HIV test outcomes of sexual partners, family members, and high-risk associates (contacts) of CommLink index clients, by program phase, and sex and age group, Eswatini, June 2015 –September 2018.

	Contacts^a^			Sexual Partners			Family Members			High-risk Associates		
Characteristic	Tested	PLHIV^b^	New Dx	Tested	PLHIV^c^	New Dx	Tested	PLHIV^d^	New Dx	Tested^e^	PLHIV	New Dx
	n	n (%)	n (%)	n	n (%)	n (%)	n	n (%)	n (%)	n	n (%)	n (%)
**Total**	537	253 (47)	113 (21)	121	73 (60)	37 (31)	287	95 (33)	43 (15)	129	85 (66)	33 (26)
**Phase**												
I (06/2015-03/2017)	193	52 (27)	30 (16)	53	30 (57)	17 (32)	140	22 (16)	13 (9)	--	--	--
II (04/2017-09/2018)	344	201 (58)	83 (24)	68	43 (63)	20 (29)	147	73 (50)	30 (20)	129	85 (66)	33 (26)
**Sex**												
Male	261	111 (43)	53 (20)	59	32 (54)	15 (25)	135	42 (31)	21 (16)	67	37 (55)	17 (25)
Female	275	142 (52)	60 (22)	62	41 (66)	22 (35)	151	53 (35)	22 (15)	62	48 (77)	16 (26)
**Age group (years)**												
<15	101	17 (17)	11 (11)	0	0 (0)	0 (0)	100	17 (17)	11 (11)	1	0 (0)	0 (0)
15–24	92	48 (52)	26 (28)	19	11 (58)	9 (47)	49	19 (39)	8 (16)	24	18 (75)	9 (38)
25–34	175	105 (60)	40 (23)	52	34 (65)	12 (23)	74	36 (49)	15 (20)	49	35 (71)	13 (27)
>34	169	83 (49)	36 (21)	50	28 (56)	16 (32)	64	23 (36)	9 (14)	55	32 (58)	11 (20)

Contacts, sexual partners, non-partner family members, or high-risk associates of CommLink index clients; family members excludes sexual partners of CommLink clients; high-risk associates, friends, coworkers, acquaintances, etc. known by CommLink clients to have defaulted from HIV care or who might benefit from HIV testing; PLHIV, person living with HIV who did not receive HIV care, including ART, in the prior 90 days; ART, antiretroviral therapy; New Dx, new HIV diagnosis.

^a^Of 262 index client contacts who tested HIV-positive, 9 reported receiving HIV care in the prior 90 days and were excluded from analyses.

^b^Includes 8 clients with missing prior diagnostic status.

^c^Includes 6 clients with missing prior diagnostic status.

^d^Includes 2 clients with missing prior diagnostic status.

^e^High-risk associates of index clients were elicited and tested during CommLink Phase II only.

### Linkage services and resolved barriers to care

Nearly all CommLink clients identified through ICT (n = 210) or outreach testing (n = 1,040) received recommended linkage services including treatment navigation (ICT, 95%; outreach, 95%), weekly telephone contact (ICT, 93%; outreach, 88%), and at least three total (two follow-up) face-to-face counseling sessions (ICT, 94%; outreach, 94%). Similar proportions of ICT- and outreach-identified clients had one or more barriers to care (ICT, 76%; outreach, 75%). Compared with outreach-identified clients, proportionally fewer ICT-identified clients had non-disclosure as a barrier, and proportionally more had barriers that involved being too busy with work or family responsibilities, stigma, denial of HIV infection, poor quality of HIV care, and ART side effects (Table 3). By the end of case management, peer counselors resolved proportionally more identified barriers of ICT than outreach clients overall, and for several types of barriers including perceived wellness, fearing response from or loss of partner, denial of HIV infection, and perceptions of low quality HIV care and disrespectful healthcare providers (Table 3).

**Table 3:** Number and type of total identified and resolved barriers to enrollment and retention in HIV care among CommLink clients, by route of participation, Eswatini, June 2015 –September 2018.

	Outreach Testing (n = 1,024)		Index Client Testing (n = 210)	
Enrollment and Retention Barriers	Total Barriers^a^	Resolved Barriers^b^	Total Barriers^a^	Resolved Barriers^b^
	n (%)	n (%)	n (%)	n (%)
**Total**	1,762	1,099 (62)	404	306 (76)
**Median (IQR)**	1 (0–3)	1 (0–1)	2 (1–3)	1 (0–2)
**Type**				
Too busy^c^	320 (31)	250 (78)	87 (41)	73 (84)
Non-disclosure^d^	182 (18)	--	7 (3)	--
Concerned about stigma^e^	180 (18)	131 (73)	49 (23)	38 (78)
Excessive alcohol use	158 (15)	64 (41)	35 (17)	15 (43)
Perceived wellness^f^	135 (13)	96 (71)	35 (17)	28 (80)
Fears response from or loss of partner^g^	129 (13)	82 (64)	27 (13)	20 (74)
ART has side effects or is ineffective^h^	100 (10)	81 (81)	37 (18)	31 (84)
Costs are too high^i^	99 (10)	66 (67)	18 (9)	13 (72)
Denies having HIV^j^	74 (7)	56 (76)	23 (11)	21 (91)
HIV-care providers are disrespectful	60 (6)	50 (83)	21 (10)	21 (100)
Quality of HIV care is poor^k^	53 (5)	44 (83)	16 (8)	16 (100)
Traditional medicine can prevent or cure AIDS	29 (3)	20 (69)	3 (1)	3 (100)
Prayer can prevent or cure AIDS	18 (2)	10 (56)	4 (2)	4 (100)
Other	225 (22)	149 (66)	42 (20)	23 (55)

IQR, interquartile range; ART, antiretroviral therapy. Outreach testing, CommLink clients who tested HIV-positive at businesses, high-volume street locations, and other community venues; Index client testing, CommLink clients who were partners, family members, or high-risk associates of other CommLink clients who tested HIV-positive as part of index-testing services.

^a^Barriers to care were assessed on 1,234 (99%) of 1,250 participants; % denominator = CommLink clients.

^b^Judged by peer counselors at the end of case management to not interfere with or prevent early enrollment or retention in HIV care; % denominator = identified barriers.

^c^Too busy with work, family, or other responsibilities to enroll or remain in HIV care.

^d^Did not disclose HIV status to any sexual partners or family members during case management. The number and proportion of persons for whom counselors helped clients disclose their HIV status (resolved disclosure barriers) was not measured.

^e^Fears loss of confidentiality and stigma when visiting healthcare facilities.

^f^Does not believe enrolling in HIV care and ART is needed because of perceived good health and wellbeing.

^g^Fears lack of support, violence, or separation from spouse or sexual partner.

^h^Believes ART has severe side effects or is ineffective.

^i^Believes transportation costs or costs from loss of work will be too high.

^j^Believes that the HIV test results were wrong and denies having HIV.

^k^Believes that the quality of HIV care is poor and does not trust healthcare providers.

### ART initiation outcomes

In all ART-eligibility periods combined, 1,215 (97%) clients enrolled in HIV care a median of 3 days (IQR = 1–7) after HIV diagnosis, and 1,120 (90%) initiated ART at a healthcare facility a median of 4 days (IQR = 1–10) after diagnosis (Table 4). Among ART-initiated clients, 902 (81%) received ART on the same day as their first clinic visit, and 1,051 (94%) received an antiretroviral refill a median of 14 days (IQR = 14–16) after ART initiation. Proportionally more clients initiated ART during test and treat when all clients were eligible for ART (96%) than during CD4<500 (81%) and CD4≤350 (66%) ART-eligibility periods. Of 210 ICT-identified clients, 95% initiated ART a median of 2 days (IQR, 1–7) after diagnosis, and of ART-initiated ICT clients, 98% received at least one antiretroviral refill before case management services ended (Table 4).

**Table 4:** Enrollment in facility-based HIV care, ART initiation, and antiretroviral refill outcomes, by ART-eligibility period, CommLink client characteristics, and route of participation, Eswatini, June 2015 –September 2018.

ART eligibility period and CommLink client characteristics	Total	Enrolled in	Initiated	Same-day	Received ≥1
	Clients^a^	HIV Care	ART	ART	ARV Refills
	n	n (%)^b^	n (%)^b^	n (%)^c^	n (%)^c^
**Total**	1,250	1,215 (97)	1,120 (90)	902 (81)	1,051 (94)
**ART eligibility periods**					
Jun 2015—Nov 2015 (CD4≤350)	137	127 (93)	90 (66)	50 (56)	78 (87)
Dec 2015—Sep 2016 (CD4<500)	289	285 (99)	235 (81)	152 (65)	214 (91)
Oct 2016—Sep 2018 (test and treat)	824	803 (97)	795 (96)	700 (88)	759 (95)
**Sex**					
Male	699	676 (97)	623 (89)	487 (78)	576 (92)
Female	551	539 (98)	497 (90)	415 (84)	475 (96)
**Age group (years)**					
15–24	207	202 (98)	185 (89)	152 (82)	170 (92)
25–34	541	521 (96)	474 (88)	389 (82)	446 (94)
>34	502	492 (98)	461 (92)	361 (78)	435 (94)
**Route of participation**					
**Outreach testing**^**d**^	1,040	1,012 (97)	920 (88)	722 (78)	855 (93)
**Index client testing**^**e**^	210	203 (97)	200 (95)	180 (90)	196 (98)
Partner	64	64 (100)	62 (97)	56 (90)	61 (98)
Family member	67	65 (97)	65 (97)	54 (83)	63 (97)
High-risk associate	79	74 (94)	73 (92)	70 (96)	72 (99)

Enrollment in HIV care, ART-initiation, same-day ART, and antiretroviral refills were abstracted from client medical records. ART, antiretroviral therapy; enrollment in HIV care, at least one documented visit at a healthcare facility to receive HIV medical care; same-day ART, received ART at the first HIV medical care visit; ARV, antiretroviral; received ≥1 ARV refills, received at least one antiretroviral refill before case management services ended; test and treat, eligible for ART regardless of CD4 count.

^a^Tested HIV-positive in community settings, reported not receiving HIV care in the prior 90 days, including ART, and consented to receive follow-up linkage case management services.

^b^% denominator = all clients.

^c^% denominator = clients who initiated ART.

^d^CommLink clients who tested HIV-positive at work sites, social venues, high-volume locations, or other community venues. During test and treat, of 631 outreach-tested clients, 610 (97%) initiated ART, 532 (84%) received same-day ART, and 578 (92%) received at least one antiretroviral refill before case management services ended.

^e^Partners, family members, or high-risk associates of other CommLink clients who were contacted and provided HIV testing services. During test and treat, of 193 index-tested clients, 185 (96%) initiated ART, 169 (88%) received same-day ART, and 181 (94%) received at least one antiretroviral refill before case management services ended.

## Discussion

Of 537 persons who received ICT through CommLink, a community-based, peer-delivered linkage case management program in Eswatini, nearly half (47%) were HIV-positive and not currently on ART, including 17%, 42%, 60%, and 66% of tested children, non-partner adult family members, sexual partners, and high-risk associates of CommLink clients, respectively. By expanding ICT for adult non-partner family members and high-risk associates, CommLink increased the yield of identifying PLHIV in need of ART from 27% in Phase I to 58% in Phase II, and identified an additional 163 PLHIV in need of ART who otherwise would not have been identified if services were restricted for only biological children and sexual partners. Of eligible ICT-identified PLHIV, nearly all participated in CommLink and received linkage case management services, and 95% initiated ART at healthcare facilities, most of whom within a few days of diagnosis. Findings from CommLink suggest that in Eswatini, expanding the scope of ICT to include all non-partner adult family members and high-risk associates, combined with providing peer-delivered linkage case management, can substantially improve case finding and ensure nearly all identified PLHIV initiate ART soon after diagnosis.

The ICT yield in new HIV diagnoses among sexual partners (31%) and children (11%) of CommLink clients is consistent with most index-testing studies in sub-Saharan Africa [4–16]. Because Eswatini surpassed 90% diagnostic coverage among all PLHIV during CommLink, we were not surprised that approximately half of identified HIV-positive sexual partners in need of ART had been previously diagnosed, and that the percentage of prior diagnoses among all identified PLHIV increased from Phase I (42%) to Phase II (59%) [23]. As countries approach near-universal diagnostic coverage among PLHIV, index-client testing and facilitated linkage services might contribute more to improving ART than diagnostic coverage by identifying and helping previously diagnosed clients re-enroll in HIV care and initiate or re-initiate ART. If not measured, HIV testing programs should consider expanding ICT indicators to capture this potentially important contribution [4–16].

Excluding social-network testing (in which a form of respondent-driven sampling is used to test members of social networks), we did not find any studies in sub-Saharan Africa that evaluated HIV testing and ART-initiation outcomes among high-risk friends and acquaintances of index clients [27]. Notably, testing high-risk associates of CommLink clients had the highest yield (66%) in identifying PLHIV in need of ART, over half (61%) of whom had been previously HIV diagnosed. Subject to confirmation elsewhere, elicitation and testing of consenting high-risk associates of index clients might be a promising strategy to help improve case finding and ART coverage among PLHIV.

Elicitation and testing of social contacts of index clients has been a longstanding practice of sexually transmitted disease control programs in the United States, particularly in controlling syphilis outbreaks [28]. In several cluster investigations, elicitation and HIV testing of social contacts of index clients has also been successful in identifying previously undiagnosed heterosexual men and women, and men who have sex with men [28–30]. Expanding ICT for high-risk associates and adult family members may also be an effective strategy to identify and initiate ART for high-priority populations [1–3, 23]. Among ICT-identified PLHIV in Phase II, 73% of adults aged 15–24 years and men were either non-partner adult family members or high-risk associates.

To optimize prevention of HIV transmission and AIDS-related morbidity and mortality, HIV testing programs need to achieve near-universal early ART initiation among all PLHIV, including those identified by index clients [1–3, 23]. In six of eight studies that reported linkage outcomes, however, only 46%-76% of ICT-identified PLHIV were linked to HIV care when provided a referral but no other linkage service, consistent with many studies of PLHIV who are diagnosed in community settings [8–13, 19, 20]. Barriers to care that might help explain low linkage rates in these studies were not reported [8–13].

Among CommLink clients, excluding non-disclosure of HIV status, similar or greater proportions of ICT- than outreach-identified clients had well-known barriers to HIV care such as being too busy with work or family responsibilities, fearing stigmatization, denial of HIV infection, and concerns about ART side effects and poor quality of HIV care [17, 18, 31, 32]. Our findings support the potential vulnerability of PLHIV to barriers following index testing, underscoring the need for ART linkage services [8–13, 17–20]. Notably, two ICT programs that provided follow-up linkage services for HIV-positive clients, including community tracing, escort, and treatment navigation by community healthcare workers or peers, achieved nearly the same ART initiation rates (89%-92%) as CommLink [15, 16].

Providing recommended linkage services such as peer-delivered, linkage case management, escort, and treatment navigation may be particularly important for many ICT-diagnosed PLHIV who were not seeking care when tested and may not have been prepared to enroll early in HIV care on their own following diagnosis [1–3, 17–25]. Beginning at the point of diagnosis, CommLink peer counselors used their personal experiences living with HIV to help clients overcome real and perceived barriers to care by providing psychosocial and informational support, testifying about how they overcame their own barriers to care, correcting misperceptions about HIV and ART, and staying with their clients during at least the first clinic visit to ensure they were comfortable and knowledgeable about receiving care at their referral facility. Notably, peer counselors were particularly effective in helping their ICT-identified clients resolve nearly all barriers involving denial of HIV infection, and perceptions of ART side effects, poor-quality of HIV care, and disrespectful healthcare providers.

Non-disclosure of HIV status is a particularly important barrier to enrollment and retention in HIV care, and testing partners and family members [17, 31–34]. Encouragingly, nearly all ICT-identified clients disclosed their status to a partner or family member, and disclosure to and elicitation of partners or family members increased from Phase I to II. Nonetheless, in Phase II only 41% of clients had ≥1 partners, family members, or associates tested through CommLink. CommLink ICT services were implemented as PEPFAR and WHO index-testing recommendations and guidance documents were being developed and disseminated [2, 3]. Greater uptake of ICT among sexual partners might have been achieved if CommLink peer counselors received training on all recommended partner elicitation and notification strategies. Our finding on high yield of new diagnoses but relatively low uptake of ICT by partners underscores an important area for future research to identify how partner-notification and ICT services might be better integrated and improved as part of peer-delivered, linkage case management.

Findings in this report are subject to at least five limitations. First, ICT outcomes are restricted to those contacts who were successfully reached and consented to test through CommLink. Our forms did not measure the number of contacts on whom tracing was initiated, and of these, contacts reached and offered HTS but decided not to test through CommLink, or decided to test at healthcare facilities or elsewhere. Second, ICT-relationship categories were based on index-client self-report, and were not routinely assessed and confirmed of partners, family members, and high-risk associates. It may be true, for example, that *associates* could have been used by some index clients to identify other sex partners whom they wished to keep anonymous. As noted elsewhere, however, an expected benefit of expanding ICT for social contacts is to allow index clients an opportunity to help test and treat partners who they do not want to identify by name [28, 30]. Third, although no reports of adverse events were received, gender-based violence and harm associated with testing partners and family members was not measured and is unknown. Having faced and overcome similar disclosure barriers as their clients, CommLink peer counselors were keenly sensitive to the potential adverse consequences of disclosure, and they worked closely with their clients ensuring that testing was offered to partners and family members only when it was safe and appropriate. Fourth, peer counselors used their own judgement in determining when identified barriers were resolved. Although the validity of their judgement is unknown for each barrier, as reported elsewhere, clients who were not linked to care had more total identified and unresolved barriers of nearly all types than those who were linked [25]. Finally, clinical data abstracted from patient healthcare cards is subject to documentation and data abstraction errors. However, in 21 healthcare facilities, senior investigators audited medical records of 408 (34%) CommLink clients and found no discrepancies on recorded enrollment in care, ART initiation, and antiretroviral refill outcomes.

## Conclusions

Findings from CommLink suggest that in high HIV-prevalence settings, expanding ICT for adult non-partner family members and high-risk associates, and providing all clients with linkage case management services, might increase the ICT yield of identifying PLHIV in need of ART, maintain a high yield of new HIV diagnoses, and ensure that nearly all contacts initiate ART within a few days of diagnosis. Notably, in Eswatini that was on the verge of achieving 95-95-95 during CommLink, two thirds of tested high-risk associates were HIV-positive and out-of-care, of whom 61% had been previously HIV diagnosed. High HIV-prevalence counties in sub-Saharan Africa striving to achieve ≥90% ART coverage among all PLHIV should consider expanding ICT services for adult non-partner family members and high-risk associates, and routinely provide peer-delivered linkage case management to all consenting HIV-positive contacts.

## Supporting information

S1 File CommLink data on eligibility and participation, index client testing, linkage services, barriers to HIV care, and enrollment in HIV care and ART initiation. (XLSX)
